# Genetic and Pharmacological Inhibition of Metabotropic Glutamate Receptor Signalling Extends Lifespan in *Drosophila*

**DOI:** 10.1111/acel.14500

**Published:** 2025-02-12

**Authors:** Cui Guan, Abigail Otchere, Mihails Laskovs, Irene Papatheodorou, Cathy Slack

**Affiliations:** ^1^ College of Health and Life Sciences Aston University Birmingham UK; ^2^ School of Life Sciences Warwick University Coventry UK; ^3^ Earlham Institute Norwich UK

**Keywords:** ageing, *Drosophila*, metabotropic glutamate receptor, ribosome biogenesis

## Abstract

Invertebrate models have been instrumental in advancing our understanding of the molecular mechanisms of ageing. The isolation of single gene mutations that both extend lifespan and improve age‐related health have identified potential targets for therapeutic intervention to alleviate age‐related morbidity. Here, we find that genetic loss of function of the G protein‐coupled metabotropic glutamate receptor (DmGluRA) in *Drosophila* extends the lifespan of female flies. This longevity phenotype was accompanied by lower basal levels of oxidative stress and improved stress tolerance, and differences in early‐life behavioural markers. Gene expression changes in *DmGluRA* mutants identified reduced ribosome biogenesis, a hallmark of longevity, as a key process altered in these animals. We further show that the pro‐longevity effects of reduced DmGluRA signalling are dependent on the fly homologue of Fragile X Mental Retardation Protein (FMRP), an important regulator of ribosomal protein translation. Importantly, we can recapitulate lifespan extension using a specific pharmacological inhibitor of mGluR activity. Hence, our study identifies metabotropic glutamate receptors as potential targets for age‐related therapeutics.

## Graphical abstract

We show that genetic and pharmacological inhibition of the metabotropic glutamate receptor, DmGluRA extends *Drosophila* lifespan, improves stress tolerance and alters early‐life locomotion. Several markers indicative of reduced ribosomal biogenesis, a hallmark of longevity, were also detected in these flies. DmGluRA post‐transcriptionally modulates the expression of the Fragile X Mental Retardation Protein (FMRP), a regulator of ribosomal protein translation, with FMRP identified as key for DmGluRA‐dependent longevity. Our study therefore highlights metabotropic glutamate receptors as potential targets for age‐related therapeutics.

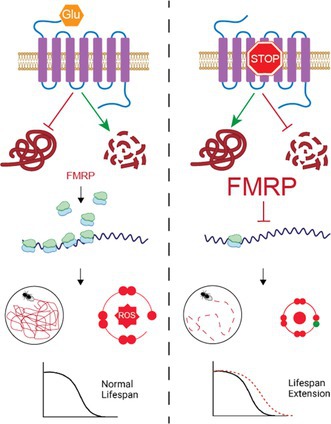

## 1 Introduction

Recent advances in geroscience research have demonstrated the plasticity of ageing. Genetic studies using model organisms including *Drosophila* have identified fundamental molecular and cellular processes that can be modified to promote healthy lifespan (López‐Otín et al. 2023). Targeting these processes with therapeutics—so‐called geroprotectors—now offers a real opportunity to translate these findings into the clinic to alleviate age‐related morbidity.

The repurposing of pharmacological agents that are already in clinical use offers an attractive route for developing novel geroprotectors as the safety profiles and clinical efficacies of these compounds are already well‐established. Of all FDA‐approved drugs, ~34% target G protein‐coupled receptors (GPCRs), the largest protein family encoded within the human genome (Hauser et al. 2017). Members of this superfamily of membrane‐bound receptors are grouped into six classes based on sequence similarity and function: Class A (rhodopsin‐like receptors), Class B (secretin family), Class C (metabotropic glutamate receptors), Class D (fungal mating pheromone receptors), Class E (cAMP receptors) and Class F (frizzled [FZD] and smoothened [SMO] receptors) (Hauser et al. 2017). They are all characterised by a seven‐transmembrane domain flanked by typically three extracellular and three intracellular loops, but the sequence similarity within these regions is poorly conserved across different receptors.

Across the six classes, GPCRs transduce extracellular signals from a diverse array of ligands, such as hormones, neurotransmitters and chemokines, to regulate key physiological processes via different intracellular signalling intermediaries including cyclic adenosine 3,5‐monophosphate (cAMP), calcium or phosphorylation of extracellular regulated protein kinases 1/2 (pERK1/2) (van Gastel et al. 2021). Ligand binding to the extracellular N‐terminus of the receptor induces a conformational change that allows the intracellular C‐terminus to interact with a heterotrimeric G protein composed of alpha, beta and gamma subunits. This facilitates the exchange of guanosine diphosphate (GDP) for guanosine triphosphate (GTP) on the alpha subunit, inducing dissociation of the alpha subunit and beta‐gamma subunits from the receptor which then interact with downstream intracellular signalling components (van Gastel et al. 2021). The nature of these signalling interactions depends on the type of G‐alpha subunit associated with the GPCR of which there are four classes: G_αs_ activates adenylyl cyclase catalysing the formation of cyclic adenosine monophosphate (cAMP) from ATP leading to activation of protein kinase A; G_αi_ primarily inhibits adenylyl cyclase to reduce cellular cAMP levels; G_αq/11_ activates phospholipase C (PLC) which catalyses the conversion of phosphatidylinositol 4,5‐bisphosphate (PIP2) to diacylglycerol (DAG) and inositol 1,4,5‐trisphosphate (IP3), leading to protein kinase C (PKC) activation and increased intracellular Ca^2+^ levels. Lastly, G_α12/13_ associates with RhoGEF to stimulate Rho GTPase activity activating several kinase cascades including RAS/MAPK (Lagunas‐Rangel 2022).

Importantly, aberrant GPCR signalling has been implicated in a diverse range of disease states that are commonly associated with increased age including type 2 diabetes, obesity, depression, cancer, Alzheimer's disease and many others (Barella et al. 2021; Gad and Balenga 2020; Thathiah and De Strooper 2011; Varney and Benovic 2024; Wong et al. 2023). More recently, GPCR activity has also been implicated in normal ageing. Genetic inactivation of GPCRs from across the classes has been shown to increase lifespan in different model organisms. For example, in *Drosophila* loss‐of‐function mutations in *Methuselah* (*Mth*) encoding a class C GPCR increase lifespan and promote stress resistance (Lin, Seroude, and Benzer 1998). Similarly, loss of the two peptide ligands for the receptor, Sun A and Sun B, can also extend fly lifespan (Cvejic et al. 2004). In the nematode, *Caenorhabditis elegans* , loss‐of‐function mutations in the serotonin receptor *SER‐1*, extends both mean and maximum lifespan (Murakami and Murakami 2007). In mice, genetic knockout of the Class A angiotensin II type 1a (AT1a) receptor extends lifespan and reduces oxidative damage across multiple organs compared to wild‐type mice (Benigni et al. 2009). Furthermore, in human population studies, particular single nucleotide polymorphisms (SNPs) that modify the activity or expression of GPCRs, including the dopamine 2 receptor and AT1a receptor, appear more frequently in people with extreme longevity such as centenarians or supercentenarians (Benigni et al. 2013; Crocco et al. 2016; Deelen et al. 2019).

Here, we show that both genetic and pharmacological inhibition of the *Drosophila* orthologue of the Class C metabotropic glutamate receptors (mGluRs) extend lifespan. *Drosophila* possesses a single functional mGluR, called DmGluRA, which shares all the structural and functional characteristics of the eight mammalian mGluRs, along with similar agonist and antagonist pharmacological profiles (Mitri et al. 2004; Parmentier et al. 1998).

In flies and mammals, mGluRs are predominantly expressed within the nervous system where they bind to the excitatory neurotransmitter, glutamate, to modulate synaptic transmission and neuronal excitability (Bruno et al. 2001). However, mGluRs are also expressed in non‐neuronal tissues such as the pancreas, lymphocytes and the intestine in mammals (Niswender and Conn 2010) and muscle, Malpighian tubules, trachea, adipose, ovarioles and testes in *Drosophila* albeit at lower levels compared to the nervous system (Li et al. 2022). Thus, mGluRs may have more diverse functions other than neural transmission.

We demonstrate that the pro‐longevity effects of DmGluRA inhibition are mediated through alterations in ribosome biogenesis, a conserved hallmark of longevity across several species (Tiku and Antebi 2018). And we present evidence that one key driver of DmGluRA‐dependent longevity is the fly homologue of the Fragile X Mental Retardation Protein (FMRP). Hence, our study demonstrates a new role for DmGluRA activity in *Drosophila* ageing and therefore presents metabotropic glutamate receptors as potential geroprotector targets.

## 2 Methods

### 2.1 Fly Strains and Husbandry

The *Drosophila* background strains, *white Dahomey* (*w* ^*Dah*^), *Wolbachia negative white Dahomey* (*w* ^*DahT*^) and *white* ^*1118*^ (*w* ^*1118*^) were used where specified. *w* ^*Dah*^ was derived by incorporation of the *w* ^*1118*^ mutation into the outbred *Dahomey* background by backcrossing. The wild‐type stock *Dahomey* was collected in 1970 in Dahomey (now Benin) and has since been maintained in large population cages with overlapping generations on a 12 h:12 h light to dark cycle at 25°C. *w* ^*1118*^ was obtained from the Bloomington Stock Centre. The *Wolbachia*‐deficient *w* ^*DahT*^ strain was generated by treating *w* ^*Dah*^ flies with Tetracycline (25 mg/mL in standard SYA food) for three generations followed by a minimum of five generations to allow for full recovery from tetracycline treatment and restoration of intestinal flora. The *DmGluRA* null mutation, *DmGluRA* ^*[112b]*^, was originally made by p‐element‐induced imprecise excision (Bogdanik et al. 2004). Other strains used in this study were: GSTD1‐GFP (transgenic GFP reporter strain which expresses GFP under the control of a 2.7 kb genomic sequence upstream of the *GSTD1* gene) (Sykiotis and Bohmann 2008) and *dFmr1* ^*[392]*^ (p‐element insertion in the *dFmr1* gene). All mutations/transgenes were backcrossed for at least six generations into the corresponding background strain before experiments were conducted.

Fly stocks were maintained, and experiments conducted at 25°C on a 12 h light/dark cycle, at constant 60% humidity, and reared on standard sugar‐yeast‐agar food (1× SYA) containing 5% (w/v) sucrose (Tate & Lyle), 10% (w/v) brewer's yeast (MP Biomedicals) and 1.5% (w/v) agar (Merck) unless otherwise stated.

Experimental flies were reared at a standardised larval density. Once emerged, adult flies were allowed to mate for 24 h and then sorted by sex under CO_2_ anaesthesia. For experiments using virgin female flies, newly eclosed female flies were collected by anaesthesia on ice.

### 2.2 Lifespan Measurements

Flies were housed at a density of 15 flies per vial and transferred into fresh vials every 2–3 days, and the numbers of deaths and censors scored. Flies were censored if they were damaged during handling, escaped or became stuck to the food. For lifespan experiments involving pharmacological inhibition of mGluR, MPEP (2‐methyl‐6‐(phenylethynyl) pyridine) (Hello Bio #HB0426, dissolved in DMSO) was added to the food at the indicated final concentrations between 0 μM (DMSO alone) and 200 μM, maintaining a final concentration of 0.2% DMSO within the fly food.

### 2.3 Female Fecundity

Ten‐day‐old female flies were transferred to fresh SYA vials, removed 24 h later and the number of eggs laid per vial were manually counted.

### 2.4 Starvation and Oxidative Stress Assays

Flies were maintained in SYA vials until 7 days post‐eclosion before transfer to either 1% (w/v) agar (Merck) in distilled water for starvation assays or SYA containing 20 mM paraquat (methyl viologen dichloride hydrate, Merck #856177) for oxidative stress assays. Deaths were recorded every 4 h, 4 times per day and flies were transferred to fresh media every 2–3 days.

### 2.5 RNA, DNA Isolation and Quantitative PCR (qPCR)

Total RNA from whole flies (10 females or 20 males) or dissected heads, thoraces and abdomens was isolated using TRIzol (Invitrogen) according to the manufacturer's instructions. For the analysis of ribosomal RNA (rRNA) expression, total RNA and DNA were extracted from the same sample using the SPINeasy DNA/RNA kit (MP Biomedicals).

RNA was converted to cDNA using random hexamers and SuperScript II Reverse Transcriptase (Invitrogen). *dFmr1* transcript expression was analysed using Taqman gene expression assays (Dm02136378_g1) and the PRISM 7000 Sequence Detection System (Applied Biosystems). Quantification was normalised to *rpl32* (Dm02151827_g1) within the log‐linear phase of the amplification curve using the comparative CT method. No differences in *rpl32* expression were detected in *dFmr1* ^*[392]*^ mutants compared to control flies. All other qPCR on cDNA or DNA was performed using 2× PrecisionPLUS qPCR master mix (PrimerDesign) on a LightCycler 480 system (Roche). Expression levels of target sequences were calculated using the relative standard curve method and normalised to the reference gene, *beta‐COP* (FBgn0008635) (Wat et al. 2020). The suitability of *beta‐COP* as a reference gene for this study was confirmed using Normfinder (https://www.moma.dk/software/normfinder). For the analysis of ribosomal RNA expression RNA/DNA ratios were calculated for each sample.

Primers for quantitative PCR were DmGluRA‐F: 5′‐AGTGTCTGTTTCTCTGCCTGG‐3′, DmGluRA‐R: 5′‐AGGCGTTGAACTCCCCTATTAT‐3′; beta‐COP‐F: 5′‐CTCTCCGAAAATGGACTTGG‐3′, beta‐COP‐R: 5′‐GACACCGAGTTCCGTCAAAT‐3′; pre‐rRNA ITS‐F: 5′‐TTAGTGTGGGGCTTGGCAACCT‐3′, pre‐rRNA ITS‐R: 5′‐CGCCGTTGTTGTAAGTACTCGCC‐3′; pre‐rRNA ETS‐F: 5′‐GTTGCCGACCTCGCATTGTTCG‐3′, prer‐RNA ETS‐R: 5′‐CGGAGCCAAGTCCCGTGTTCAA‐3′; pre‐rRNA 18S‐F: 5′‐TGTAGCCTTCATTCATGTTGGCAG‐3′, pre‐rRNA 18S‐R: 5′‐ACCAACAGGTACGGCTCCAC‐3′; pre‐rRNA 28S‐F: 5′‐CCTGCCGAAGCAACTAGCCCTT‐3′, pre‐rRNA 28S‐R: 5′‐CCATGCAGGCTTACGCCAAAC‐3′.

### 2.6 Western Blot Analysis

Five whole adult flies were lysed in 100 μL Laemmli buffer containing 100 mM DTT (1,4‐Dithiothreitol) and proteins were denatured at 85°C for 10 min. ~20 μg of proteins were separated using 10% SDS–PAGE gels and transferred onto a 0.45‐μm nitrocellulose membranes.

Primary antibodies used were anti‐GFP (diluted 1:10,000; Abcam #ab6556) and anti‐Fmr1 (diluted 1:1000; Abcam #ab10299). Anti‐beta actin (diluted 1:10,000; Abcam #ab8224) was used as a loading control. HRP‐conjugated secondary antibodies—either goat anti‐rabbit IgG (1:10,000 dilution; Abcam #ab6721) or goat anti‐mouse IgG (1:10,000 dilution; Abcam #ab6789)—were used depending on the corresponding species of the primary. Blots were developed using the ECL detection system (Bio‐Rad). Signals were captured using a G:BOX gel documentation system (SynGene) and band intensities were quantified using Fiji in ImageJ (Schindelin et al. 2012).

### 2.7 Puromycin Incorporation Assays

Individual flies were cut open along the ventral midline from thorax to abdomen in ice‐cold Schneider's media (Merck #S0146). Each fly was then transferred to 1.0 mL of Schneider's media prewarmed to 25°C containing 10 μg/mL puromycin (Merck #P9620) and incubated for 30 min at room temperature. Flies were then lysed in 20 μL Laemmli buffer containing 100 mM DTT and homogenates incubated at 85°C for 10 min. *w* ^*Dah*^ and *DmGluRA* ^*[112b]*^ mutant flies were processed side‐by‐side as paired samples. 10 μL of the protein extract were loaded onto 4%–12% gradient gels (Bio‐Rad) before Western blotting using anti‐puromycin antibody (diluted 1:25,000, Millipore #12D10). Anti‐beta tubulin antibody (diluted 1:10,000; Abcam #ab108342) was used as the loading control. The intensities of anti‐puromycin staining between 15 and 165 kDa were quantified relative to tubulin.

### 2.8 Immunohistochemistry of Fly Guts

Midguts were dissected from 10‐day‐old flies in ice‐cold PBS and transferred directly to cold 4% (v/v) methanol‐free formaldehyde in PBS. Guts were washed twice for 10 min each in PBST (0.2% (v/v) Triton X‐100/PBS) at room temperature, blocked in 5% (v/v) normal goat serum/PBST for 1 h at room temperature and then incubated in primary antibody (1:200 rabbit anti‐Fibrillarin, Abcam #ab5821) overnight at 4°C. Guts were washed four times for 15 min each in PBST and then incubated in secondary antibody (1:500 goat anti‐rabbit AlexaFluor 488, Abcam #ab150077) for 2 h at room temperature before washing and mounting in Fluoroshield containing DAPI (Merck). Guts were imaged using a Leica SP8 Lightning confocal microscope and LASX software. Fibrillarin staining for the nucleolus and DAPI staining for the nucleus were manually traced and measured using Fiji within ImageJ from randomly selected enterocytes located within the posterior midgut at their widest diameter. Representative images were processed in LASX (Leica) and Adobe Photoshop (Adobe) for publication.

### 2.9 Negative Geotaxis Assays

Flies were maintained in SYA vials (15 flies per vial) and tipped using *Drosoflippers* (www.drosoflipper.com). Climbing ability was assessed in empty vials placed on either side of the *Drosoflipper* by banging the flies to the bottom of the vial and video recording for 40 s. Still images from each video were captured after 15 s and analysed in Fiji in ImageJ. Assays were recorded at the same hour each weekday in each week for 8 weeks using the same cohort of flies throughout.

### 2.10 Exploratory Walking Assays

Individual flies were aspirated into separate 4 cm diameter/1 cm height circular plastic arenas. Flies were rested for 1 min before 15‐min video recordings were taken. Videos were taken every other week for 9 weeks. Videos were analysed with Ethovision XT video tracking software (Noldus). Fresh flies were used at each recording time point.

### 2.11 RNA‐Sequencing

Total RNA was extracted from 10‐day‐old flies using TRIzol as described before. cDNA libraries were prepared and sequencing (150 bp paired‐end) performed by Novogene Co. Ltd. (UK) using Illumina NovaSeq platforms. Sequences were quality checked by FastQC and reads below the minimum quality threshold *Q* < 10 were discarded. Orphaned reads after filtering were also discarded. Reads were mapped to the Berkeley *Drosophila* Genome Project (BDGP) *Drosophila melanogaster* genome assembly release 6 (Ensembl release v51) using HISAT2 version 2.1.0. Reads that were uniquely aligned to annotated genes were counted with FeatureCounts version 1.6.2. Differential expression analysis was performed using DESeq2 version 1.10.1. Significance was determined using the standard DESeq2 FDR cutoff of adjusted *p* < 0.05. Gene Ontology (GO) term enrichment analysis was performed using g:Profiler (https://biit.cs.ut.ee/gprofiler/gost).

### 2.12 Statistics and Reproducibility

Sample sizes were determined based on previous publications using similar experimental strategies and are indicated within the figure legends. No specific methods were used to randomly allocate samples to groups. Data distributions were assumed to be normal, but this was not formally tested. No data were excluded from the analysis. Data collection and analysis were carried out in an unblinded fashion unless otherwise stated. Statistical analyses were mainly performed in R studio (R v3.5.5), Prism (v10.0, Graphpad) or Jmp (v14, SAS). Survival data were analysed using Log‐rank test in Excel (Microsoft) or Cox proportional hazards tests in R using the ‘survival’ package (https://CRAN.R‐project.org/package=survival). Other statistical tests used are indicated in the figure legends.

## 3 Results

### 3.1 Mutation of the Metabotropic Glutamate Receptor (*DmGluRA*) in *Drosophila* Extends Female Lifespan

**FIGURE 1:**
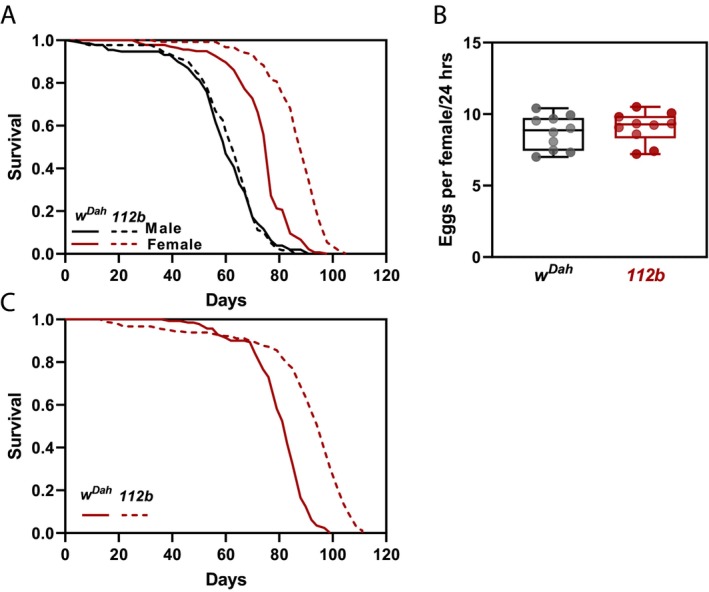
Mutation of the metabotropic glutamate receptor (DmGluRA) in *Drosophila* extends female lifespan. (A) Survival of *DmGluRA* ^*[112b]*^ homozygous mutants compared to *w* ^*Dah*^ controls (*w* ^*Dah*^ males: *n* = 127 dead/8 censored; *DmGluRA* ^*[112b]*^ males: *n* = 117 dead/18 censored; *p* = 0.8, Log‐rank test. *w* ^*Dah*^ females: *n* = 136 dead/6 censored; *DmGluRA* ^*[112b]*^ females: *n* = 117 dead/19 censored; *p* = 4.6 × 10^−23^, Log‐rank test). (B) Number of eggs laid per fly over 24‐h for 10‐day‐old females. Box‐and‐whisker plot represents minimum, 25%, median, 75% and maximum. Individual data points represent average number of eggs laid per fly per vial (15 flies per vial, *n* = 10 vials). No significant difference: unpaired *t*‐test, *p* = 0.51. (C) Survival of virgin female *DmGluRA* ^*[112b]*^ homozygous mutants compared to virgin female *w* ^*Dah*^ controls (*w* ^*Dah*^ virgin females: *n* = 133 dead/19 censored; *DmGluRA* ^*[112b]*^ virgin females: *n* = 173 dead/14 censored; *p* = 1.4 × 10^−25^, Log‐rank test).

To determine a role for metabotropic glutamate receptors in ageing, we examined the effects of null mutation of the single *Drosophila* orthologue, *DmGluRA* ^*[112b]*^, on lifespan. This deletion extends from the *DmGluRA* promoter into the coding sequence and homozygotes do not express detectable levels of the receptor and so are considered null mutants (Bogdanik et al. 2004). Female flies homozygous for the *DmGluRA* ^*[112b]*^ deletion lived significantly longer than controls, but similar lifespan benefits were not apparent for males (Figure 1A). We observed a comparable lifespan extension for *DmGluRA* ^*[112b]*^ mutants in five independent cohorts at different times (Figure S1). Furthermore, we were able to fully reproduce the longevity effects of *DmGluRA* ^*[112b]*^ homozygotes in an independent genetic background, *w* ^*1118*^, as well as in the absence of the endosymbiont, *Wolbachia pipientis* (Figure S2). Taken together, mutation of *DmGluRA* extended the lifespan of female flies irrespective of genetic background or *Wolbachia* status. As we did not observe any effects on male lifespan, we focused on understanding the mechanism by which DmGluRA activity impacts on longevity in females.

Reproductive trade‐offs are often documented in long‐lived *Drosophila* where several interventions that extend lifespan also reduce female fecundity (Flatt 2011). We therefore examined the number of eggs laid by 10‐day‐old female flies but found no significant differences between mutant females and controls (Figure 1B). We also examined the lifespan of *DmGluRA* ^*[112b]*^ virgin females and observed a similar extension of lifespan as mated females (Figure 1C). Thus, mutation of *DmGluRA* extends female lifespan irrespective of mating and reproductive capacity.

### 3.2 *DmGluRA* Shows Both Age‐ and Sex‐Dependent Changes in Expression

**FIGURE 2:**
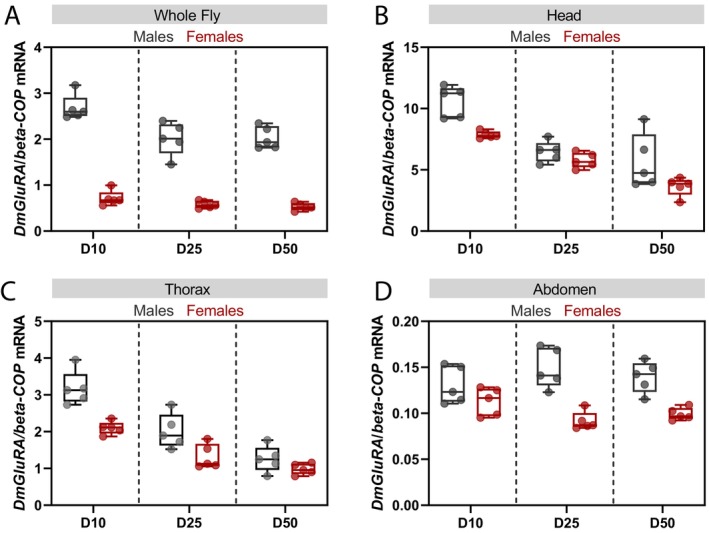
*DmGluRA* transcripts show sex‐ and age‐dependent effects on expression. Relative DmGluRA transcript levels measured by qRT‐PCR for total RNA extracted from (A) whole flies (*n* = 5 biologically independent samples, effect of age *p* = 0.00039, sex *p* < 10^−4^, sex‐by‐age interaction *p* = 0.02975, LM); (B) isolated heads (*n* = 5 biologically independent samples, effect of age *p* < 10^−4^, sex *p* = 0.001, sex‐by‐age interaction not significant, LM); (C) isolated thoraces (*n* = 5 biologically independent samples, effect of age *p* < 10^−4^, sex *p* = 0.00005, sex‐by‐age interaction not significant, LM), and (D) isolated abdomens (*n* = 5 biologically independent samples, effect of age and sex not significant, sex‐by‐age interaction *p* = 0.036, LM). Box‐and‐whisker plots represent minimum, 25%, median, 75% and maximum with values for individual biological replicates overlaid as points.

Sexual dimorphism in phenotypic outputs can often be mediated by sex differences in gene expression (Bain et al. 2021). As the pro‐longevity effects of loss of DmGluRA activity were exclusively observed in females and not in males, we examined control flies for sex‐biased expression of *DmGluRA* transcripts. Flies were collected at three different points (10‐, 25‐ and 50‐ days of age) to also examine if *DmGluRA* expression changes with age in each sex. Using a mixed effects linear model, we found that *DmGluRA* mRNA expression is indeed sexually dimorphic with significantly higher expression in males than females at all three ages (*p* < 0.0001, Figure 2A). We also observed an age‐dependent decrease in *DmGluRA* expression during ageing (*p* = 0.0004, Figure 2A) but this was dependent on sex (sex‐by‐age interaction *p* = 0.0297, Figure 2A).

Many genes show age‐related changes in their expression and for some genes these transcriptional changes are observed in specific tissues (Frenk and Houseley 2018). We therefore examined changes in *DmGluRA* expression during ageing in the three different body segments of the fly—head, thorax and abdomen. Sexually dimorphic expression of *DmGluRA* transcripts was found only in the heads and thoraces again across all three ages, with significantly higher *DmGluRA* expression in males (Figure 2B,C). We also detected a similar decrease in *DmGluRA* expression with age in the heads and thoraces of both males and females as observed in whole flies (Figure 2B,C). However, while *DmGluRA* expression levels were comparable in the abdomens of younger males and females, we identified a significant age‐by‐sex effect on *DmGluRA* expression in this body segment (Figure 2D) suggesting that in the abdomen of the fly, *DmGluRA* expression shows a more pronounced age‐dependent decrease in females than in males. It is important to note that *DmGluRA* expression varied across the different body segments with most expression observed within heads, moderate expression within the thorax and lowest expression in the abdomen. Together, these data show that *DmGluRA* transcript expression is higher in males than in females but decreases with age in both sexes.

### 3.3 *DmGluRA* Mutants Are Stress Resistant

**FIGURE 3:**
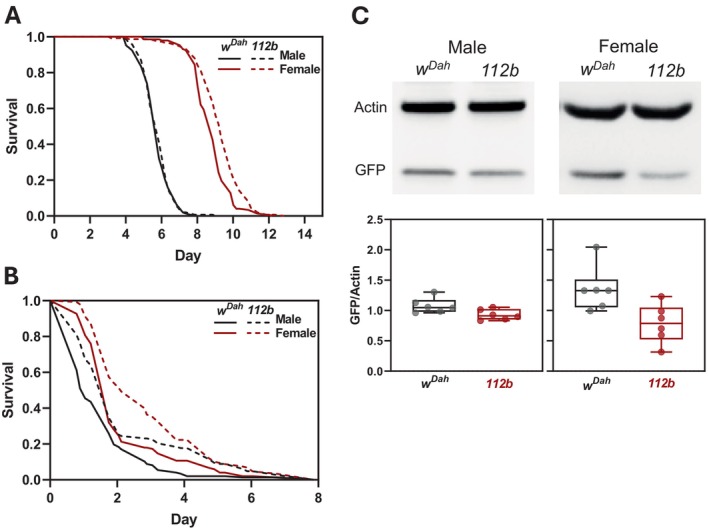
*DmGluRA* mutants are resistant to starvation and oxidative stress. (A) Survival of *DmGluRA* ^*[112b]*^ homozygous mutants compared to *w* ^*Dah*^ controls during starvation. Males (black lines): *w* ^*Dah*^ *n* = 146 dead/0 censored, *DmGluRA* ^*[112b]*^ *n* = 146 dead/0 censored; *p* = 0.73, Log‐rank test; females (red lines): *w* ^*Dah*^ *n* = 148 dead/0 censored, *DmGluRA* ^*[112b]*^ *n* = 150 dead/0 censored; *p* = 2.4 × 10^−5^, Log‐rank test. (B) Survival of *DmGluRA* ^*[112b]*^ homozygous mutants compared to *w* ^*Dah*^ controls in the presence of 20 mM paraquat. Males (black lines): *w* ^*Dah*^ *n* = 147 dead/0 censored, *DmGluRA* ^*[112b]*^ *n* = 147 dead/0 censored; *p* = 5.1 × 10^−5^, Log‐rank test; females: *w* ^*Dah*^ *n* = 150 dead/0 censored; *DmGluRA* ^*[112b]*^ *n* = 148 dead/0 censored; *p* = 4.2 × 10^−5^, Log‐rank test. (C) Representative Western blots and quantification of GST::GFP expression in 10‐day‐old male and female *w* ^*Dah*^ controls and *DmGluRA* ^*[112b]*^ homozygous mutants normalised to actin. Box‐and‐whisker plots represent minimum, 25%, median, 75% and maximum with values for individual biological replicates overlaid as points (*n* = 6 biologically independent samples, males *p* = 0.038, females *p* = 0.018, *t*‐test).

Interventions that extend fly lifespan are often associated with resistance to various stresses (Vermeulen and Loeschcke 2007). We therefore tested *DmGluRA* ^*[112b]*^ mutant flies for survival under starvation and with exposure to paraquat, an oxidative stress inducer. For starvation assays, 7‐day‐old flies were transferred to agar‐only media with no nutritive value. *DmGluRA* ^*[112b]*^ homozygous females showed a significant increase in survival during starvation compared to controls (Figure 3A, median survival +8%, *p* = 2.4 × 10^−5^) but no differences were observed in *DmGluRA* ^*[112b]*^ homozygous males (Figure 3A). To characterise the survival response of *DmGluRA* ^*[112b]*^ mutants to oxidative stress, 7 day‐old flies were transferred to food containing 20 mM paraquat. Both male and female flies homozygous for the *DmGluRA* ^*[112b]*^ deletion showed significantly increased survival under oxidative stress compared to controls (Figure 3B, for males, median survival +50%, *p =* 5.1 × 10^−5^; for females, median survival +40%, *p =* 4.2 × 10^−5^). This increase in survival under oxidative stress induced by paraquat was associated with reduced basal expression of a GFP reporter for the oxidative stress response gene, *glutathione S‐transferase* (Figure 3C).

### 3.4 Loss of *DmGluRA* Impacts on Neuromuscular Health

**FIGURE 4:**
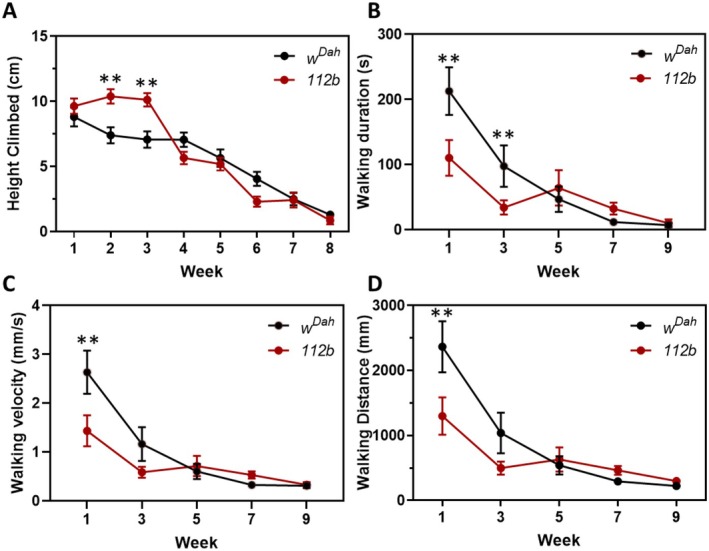
Loss of *DmGluRA* impacts on neuromuscular health. (A) Climbing/negative geotaxis assay. Each point shows the mean height climbed ± SEM during negative geotaxis assays for *w* ^*Dah*^ control and *DmGluRA* ^*[112b]*^ homozygous females. At the start of the experiment (week 1) *n* = 68–69 flies. As the experiment progressed flies began to die so at week 8, *n* = 13–39. No significant effect of genotype, age *p* < 2 × 10^−16^, genotype‐by‐age interaction *p* = 0.0002, LM. (B–D) Exploratory walking duration, velocity and walking distance of *w* ^*Dah*^ control and *DmGluRA* ^*[112b]*^ homozygous females. Each point shows mean ± SEM (*n* = 23–28, two batches). There was a significant effect of genotype (*p* < 0.001), age (*p* < 0.001) and a significant genotype‐by‐age interaction (*p* < 0.01) for all three measured parameters, mixed effects linear model (with batch as a random effect). **Post hoc *t*‐test.

A decline in neuromuscular function is a common aetiology of ageing across many different species including *Drosophila* (Rhodenizer et al. 2008). As *DmGluRA* transcripts are highly expressed within the nervous system, we examined long‐lived *DmGluRA* ^*[112b]*^ females for age‐related functional changes in the neuromuscular system. We first examined negative geotaxis, an induced escape response that declines with age, measured by the ability of flies to climb a vertical surface. Interestingly, we found that young *DmGluRA* ^*[112b]*^ mutant flies during the first 3 weeks of adulthood climbed significantly higher than control flies (Figure 4A). However, we observed no differences in their age‐related decline in climbing ability (Figure 4A).

We also analysed neuromuscular function using an exploratory walking assay (Ismail et al. 2015), a spontaneous, yet complex behaviour observed when flies are placed into a new environment.

Interestingly, we found that walking duration, velocity and distance were all significantly lower in *DmGluRA* ^*[112b]*^ mutant flies during early life compared to controls (Figure 4B–D). However, at later ages there were no significant differences between *DmGluRA* ^*[112b]*^ mutants and controls in these three behavioural parameters. Thus, loss of DmGluRA activity has beneficial effects on climbing ability but reduces certain parameters of exploratory walking behaviour during early life.

### 3.5 *DmGluRA* Mutation Is Associated With Changes in Ribosomal RNA Production and Processing

**FIGURE 5:**
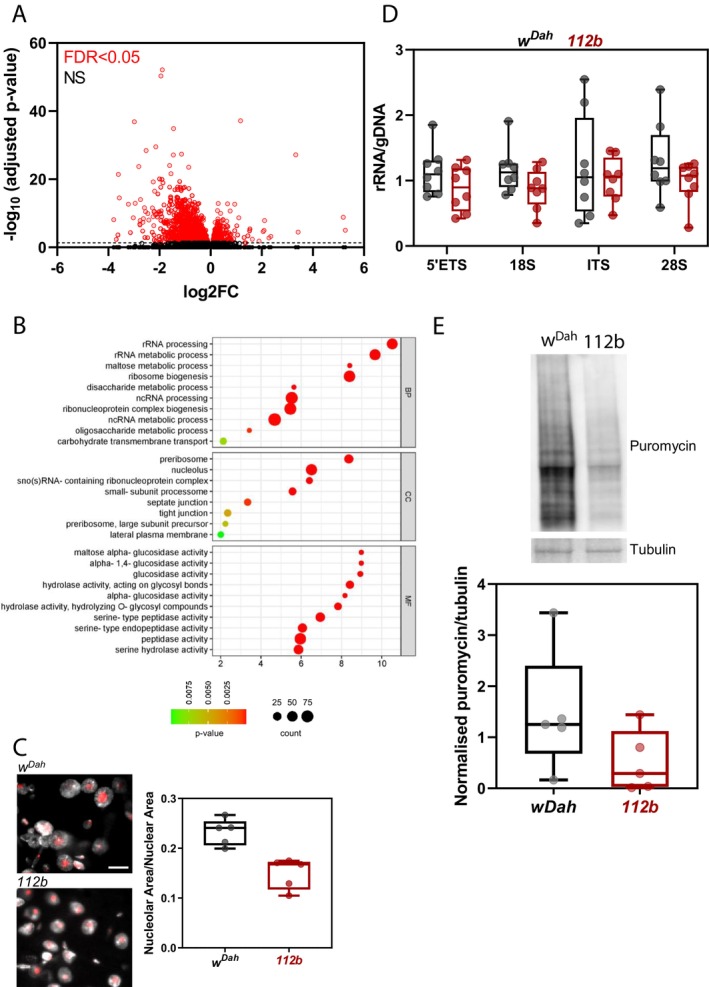
Loss of *DmGluRA* activity is associated with changes in ribosomal RNA production and processing. (A) Volcano plot of differential gene expression in *DmGluRA* ^*[112b]*^ mutants versus *w* ^*Dah*^ controls (FDR 0.05%) shown in red. Nine hundred and eighty‐three genes show upregulated expression and 1288 genes show downregulated expression. Each dot represents an individual gene. Horizontal black dotted line denotes adjusted *p*‐value threshold of −log_10_(0.05). (B) Gene ontology (GO) analysis of the differentially expressed genes in *DmGluRA* ^*[112b]*^ mutants. Bubble plot shows the top GO terms (FDR < 0.05) for a ranked list of all differentially expressed genes. (C) Representative images of nucleoli (Fibrillarin immunostaining) in the midgut enterocytes of *w* ^*Dah*^ and *DmGluRA* ^*[112b]*^ homozygous mutant flies (scale bar, 10 μm) and quantification of nucleolar size in midgut enterocyte nuclei (determined as each nucleolar area divided by each nuclear area). Box‐and‐whisker plots represent minimum, 25%, median, 75% and maximum with values for biological replicates overlaid as points (average nucleolar area/nuclear area of 10 cells per fly, *n* = 5 flies, *p* = 0.0019, *t*‐test). (D) Ratio of RNA to DNA for the sequences present in pre‐rRNA‐rDNA (*n* = 8 biologically independent samples, effect of genotype *p* = 0.0199, but no significant effect of the target sequence or interaction, LM). (E) Representative Western blot and quantification of puromycin incorporation normalised to tubulin. Box‐and‐whisker plots represent minimum, 25%, median, 75% and maximum overlaid with individual data points (*n* = 5 individual flies, *p* = 0.0433, *t*‐test).

To identify candidate mechanisms whereby loss of DmGluRA extends lifespan, we compared genome‐wide transcriptional changes in *DmGluRA* ^*[112b]*^ mutants to controls using RNA‐sequencing. We identified 2271 genes that were differentially expressed (10% FDR) with 983 genes showing increased expression and 1288 genes showing decreased expression in *DmGluRA* ^*[112b]*^ mutants compared to control flies (Figure 5A). Using g:Profiler, we identified Gene Ontology (GO) annotations enriched within a ranked list of these differentially expressed genes (DEGs) and observed an overrepresentation of genes linked to ribosome biogenesis (Figure 5B).

Ribosomal RNA production and ribosome assembly take place in the nucleolus within the nucleus of the cell. Small nucleolar size is indicative of reduced ribosome biogenesis and is a cellular hallmark of longevity in several species (Tiku and Antebi 2018). We therefore analysed nucleolar size by staining for the nucleolar marker, fibrillarin, within the enterocytes (ECs) of the *Drosophila* midgut where *DmGluRA* transcripts are reportedly expressed across all regions albeit at low levels (Dutta et al. 2015). We found that *DmGluRA* ^*[112b]*^ mutants showed a significant reduction in nucleolar area relative to nuclear area within ECs compared to controls (Figure 5C).

We also examined whether production of ribosomal RNAs was altered in *DmGluRA* ^*[112b]*^ mutants using qPCR to determine the relative RNA to DNA abundance of pre‐rRNA as well as for the mature 18S and 28S rRNA. We observed a 27% overall reduction in total rRNA/rDNA ratio in *DmGluRA* ^*[112b]*^ mutants compared to controls (Figure 5D). Furthermore, this reduction in rRNA production was associated with reduced protein synthesis as detected by puromycin incorporation (Figure 5E). Therefore, *DmGluRA* loss‐of‐function is associated with a reduction in ribosomal RNA production and protein synthesis, both of which may contribute to the longevity phenotype of *DmGluRA* ^*[112b]*^ mutants.

### 3.6 Pro‐Longevity Effects of *DmGluRA* Mutation Are Dependent on FMRP

Metabotropic glutamate receptors share an antagonistic relationship with the Fragile X Mental Retardation Protein (FMRP). FMRP is expressed from the fragile X mental retardation 1 (*FMR1*) gene, mutations in which cause Fragile X, an inheritable disease characterised by mental retardation. FMRP is an RNA‐binding protein that has a well‐established role as a translational regulator through its association with polyribosomes and direct binding to specific sequences or structures within target mRNAs (Laggerbauer et al. 2001). More recently, FMRP has been shown to also regulate ribosomal protein translation and ribosome biogenesis (Seo et al. 2022). We therefore asked whether the pro‐longevity effects of loss of DmGluRA signalling observed here were dependent on increased FMRP expression.

**FIGURE 6:**
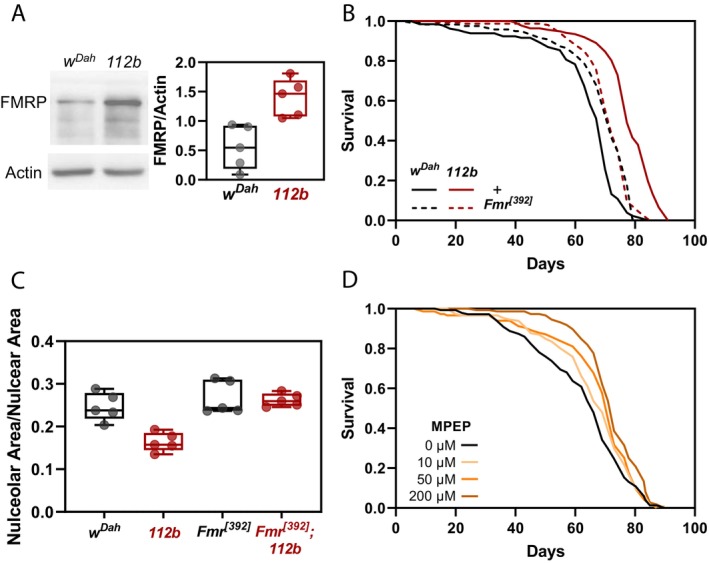
Pro‐longevity effects of reduced DmGluRA signalling are dependent on FMRP. (A) Representative Western blot and quantification of FMRP expression in 10‐day‐old female *w* ^*Dah*^ and *DmGluRA* ^*[112b]*^ homozygous mutant flies normalised to actin. Box‐and‐whisker plots represent minimum, 25%, median, 75% and maximum with data points for individual biological replicates overlaid (*n* = 5 biologically independent samples, *p* = 0.0024, *t*‐test). (B) Survival of female flies either homozygous mutant for *DmGluRA* ^*[112b]*^ alone or combined with the *Fmr* ^*[392]*^ allele. *w* ^*Dah*^ (*n* = 129 dead/4 censored), *DmGluRA* ^*[112b]*^ homozygotes (*n* = 137 dead/0 censored), *Fmr* ^*[392]*^ homozygotes (*n* = 117 dead/3 censored) and *Fmr* ^*[392]*^ *;DmGluRA* ^*[112b]*^ double mutants (*n* = 152 dead/2 censored; *Fmr* ^*[392]*^ vs. *Fmr* ^*[392]*^ *;DmGluRA* ^*[112b]*^, *p* = 2.798 × 10^−17^, Log‐rank test). (C) Quantification of nucleolar size in midgut enterocyte nuclei (determined as each nucleolar area divided by each nuclear area). Box‐and‐whisker plots represent minimum, 25%, median, 75% and maximum with values for biological replicates overlaid as points (average nucleolar area/nuclear area of 10 cells per fly, *n* = 5 flies, *p* < 0.0001, ANOVA). (D) Survival of *w* ^*Dah*^ female flies in the presence of DMSO alone (0 μM) or 10, 50 or 200 μM 2‐Methyl‐6‐(phenylethynyl)pyridine (MPEP), a potent pharmacological inhibitor of mGluRA (0 μM MPEP: *n* = 134 dead/11 censored; 10 μM MPEP: *n* = 143 dead/7 censored; 50 μM MPEP: *n* = 137 dead/11 censored; 200 μM MPEP: *n* = 138 dead/11 censored; 0 μM vs. 50 μM, *p* = 0.032 Log‐rank test; 0 μM vs. 200 μM, *p* = 1.36 × 10^−5^, Log‐rank test).

To validate the link between DmGluRA and FMRP in our study, we first measured FMRP levels in female *DmGluRA* ^*[112b]*^ mutant flies by Western blot and as predicted, saw elevated expression of FMRP in *DmGluRA* ^*[112b]*^ mutants compared to controls (Figure 6A). To determine whether elevated FMRP expression was required for the longevity effects of the *DmGluRA* ^*[112b]*^ mutation, we performed genetic epistasis experiments using a hypomorphic p‐element insertion, *dFmr1* ^*[392]*^, that results in reduced *dFmr1* transcript and protein expression (Figure S3). We found that while homozygosity for the *dFmr1* ^*[392]*^ allele slightly increased survival by itself, it almost completely removed the longevity effects of the *DmGluRA* ^*[112b]*^ mutation (Figure 6B). We used Cox proportional hazards (CPH) analysis with relevant a priori contrasts to confirm that the *DmGluRA* ^*[112b]*^ mutation had a significantly different impact on the lifespan of control flies compared to *dFmr* ^*[392]*^ mutants (Table S1, *p* < 2.0 × 10^−16^). This suggests that increased FMRP expression upon loss of DmGluRA is required for lifespan extension.

We next asked whether this FMRP‐dependent rescue of lifespan extension in *DmGluRA* ^*[112b]*^ mutants was also associated with the restoration of ribosome biogenesis within the nucleolus. Again, we analysed relative nucleolar size within midgut ECs using fibrillarin staining. Interestingly, we saw that the small nucleolar area relative to nuclear area observed in *DmGluRA* ^*[112b]*^ mutant ECs was fully restored to normal size in *dFmr1* ^*[392]*^ *;DmGluRA* ^*[112b]*^ double mutants (Figure 6C).

### 3.7 Pharmacological Inhibition of DmGluRA Also Extends Lifespan

2‐Methyl‐6‐(phenylethynyl) pyridine (MPEP) is a selective antagonist for the mammalian mGluR5 receptor and was shown in a previous study to also inhibit the *Drosophila* DmGluRA receptor (McBride et al. 2005). Having demonstrated genetically that loss of *DmGluRA* signalling increases lifespan in female flies, we wanted to see whether pharmacological inhibition of DmGluRA activity could achieve a similar effect. We supplemented the fly food with either vehicle alone (DMSO) or MPEP at concentrations of 10, 50 and 200 μM MPEP. No significant differences in lifespan were observed at 10 μM MPEP but we observed a dose‐dependent increase in lifespan at 50 and 200 μM MPEP compared to flies fed with vehicle alone (0 μM vs. 50 μM, median lifespan +7%, *p* = 0.032 (Log‐rank); 0 μM vs. 200 μM, median lifespan +11%, *p* = 1.37 × 10^−5^ (Log‐rank); Figure 6D). Thus, both genetic and pharmacological inhibition of DmGluRA extends lifespan in *Drosophila*.

## 4 Discussion

A growing body of work has highlighted an important role for GPCR signalling during normal ageing. However, the relationship between GPCR activity and ageing is complex depending on the GPCR under study. As such, overexpression or genetic deletion of different GPCRs in animal models produce differential effects on lifespan with the activity for some GPCRs proving beneficial for lifespan while the activity of others are life‐limiting (Lagunas‐Rangel 2022). A large proportion of GPCRs are pharmacological targets and so a better understanding of the role of specific GPCRs in ageing could offer novel routes for intervention to alleviate age‐related morbidity. Here, we present evidence that inhibition of the single orthologue of the class C metabotropic glutamate receptor, DmGluRA, extends lifespan in *Drosophila*. The relevance of this finding is further highlighted by our discovery that pharmacological inhibition of DmGluRA activity with the highly specific inhibitor, 2‐Methyl‐6‐(phenylethynyl)‐pyridine (MPEP), produces similar pro‐longevity effects. Thus, we have identified mGluR inhibitors as potential geroprotectors that could be harnessed to improve age‐related health.

We have found that loss of DmGluRA activity produces a sexually dimorphic response in the context of lifespan. As such, we only saw the pro‐longevity effects of DmGluRA inhibition in females and not in males. Moreover, we have also observed sex differences in the expression of *DmGluRA* transcripts with higher levels of *DmGluRA* gene expression in males across multiple tissues compared to females. It is not unusual for lifespan‐extending interventions to increase the lifespan of females but have limited effects in males (Austad and Fischer 2016). For example, down‐regulation of the nutrient‐sensing insulin/insulin‐like growth factor signalling pathway extends lifespan in female animals to a greater extent than in males, particularly in *Drosophila* (Austad and Fischer 2016). Sex‐biased gene expression is also common with studies reporting sex‐based variation in expression for approximately 40% of the *Drosophila* transcriptome while in mammals, sex‐biased gene expression depends on species and tissue (Huang et al. 2015; Rodriguez‐Montes et al. 2023). It is not yet clear how the differences in expression of DmGluRA between males and females may contribute to lifespan but sexual dimorphisms in gene expression, including autosomal gene expression, often contribute to sexually dimorphic phenotypes.

Loss of mGluR activity has been previously reported to reduce lifespan in *Drosophila* rather than promote longevity (Ly and Naidoo 2019). However, we note that there are several methodological differences that could account for these differences in lifespan outcomes including differences in diet. Here, we have used a diet that has been optimised for ageing studies and used extensively to characterise the longevity effects of a range of interventions (Piper and Partridge 2016). Genetic background can also significantly influence lifespan responses although we were able to fully reproduce the longevity effects of *DmGluRA* ^*[112b]*^ homozygotes in an independent genetic background, *w* ^*1118*^, as well as in the absence of the endosymbiont, *Wolbachia*. Furthermore, we could recapitulate the pro‐longevity effects of *DmGluRA* ^*[112b]*^ mutation using the pharmacological inhibitor of DmGluRA activity, MPEP. Thus, our data strongly suggest that DmGluRA inhibition produces robust lifespan extension.

Interestingly, alongside a longer lifespan, genetic inhibition of DmGluRA signalling was also associated with resistance to chronic stress, both nutritional stress in the form of starvation and oxidative stress. Indeed, *DmGluRA* mutant flies were found to have lower basal levels of an oxidative stress marker compared to controls. Improved tolerance to a variety of common stress conditions including starvation, oxidative stress and high temperature seems to be a common feature across multiple classes of GPCR whose activity impacts on animal lifespan (Lagunas‐Rangel 2022). For example, in *Drosophila* long‐lived *Mth* mutants also showed enhanced resistance to oxidative stress associated with increased Mn‐ and Cu/Zn‐superoxide dismutase activity (Cvejic et al. 2004). Similarly, in *C. elegans* , loss‐of‐function mutations in the serotonin receptor *SER‐1* extend both mean and maximum lifespan and mutant worms also show enhanced resistance to oxidative stressors (Murakami and Murakami 2007). Thus, altered stress resistance may be a common feature associated with longevity upon modulation of GPCR activity. However, enhanced stress resistance, particularly to oxidative stress, may not necessarily contribute to the pro‐longevity phenotype observed in *DmGluRA* ^*[112b]*^ mutants. Male and female homozygous mutants were both resistant to oxidative stress induced by paraquat, but only female mutants were long‐lived suggesting that these two phenotypes can be uncoupled.

Our behavioural analysis of *DmGluRA* mutants during ageing revealed that loss of DmGluRA activity predominantly impacts on parameters of neuromuscular function during early life with improved climbing ability but reduced markers of exploratory walking behaviour in young *DmGluRA* mutants compared to controls. However, at later ages these behavioural phenotypes were comparable between genotypes. Although early climbing ability does not necessarily correlate with lifespan, studies have suggested an inverse correlation between high energy behaviours and longevity (Overman et al. 2022). Thus, long‐lived mutants are expected to display reduced energy costly locomotory behaviours and idle more. The reduced exploratory walking activity of *DmGluRA* mutants during early life may therefore contribute to their longevity.

Previous studies had already established an antagonistic relationship between Group 1 mGluRs (mGluR1 and mGluR5) and the Fragile X Mental Retardation Protein (FMRP). Our current work confirms such an association as not only do we observe increased FMRP expression in *DmGluRA* mutant flies, but we have also demonstrated that the pro‐longevity effects of *DmGluRA* mutation are dependent upon FMRP. Group 1 mGluRs have been shown to regulate FMRP expression in mammals through multiple mechanisms including via CREB‐dependent transcriptional control (Wang et al. 2012) as well as through the regulation of FMRP translation and proteasomal degradation (Hou et al. 2006). We note that *dFmr1* transcripts were not identified as differentially expressed within our RNA‐seq data suggesting that the differences in FMRP protein expression in *DmGluRA* ^*[112b]*^ mutant flies are most likely due to post‐transcriptional mechanisms.

FMRP has recently been shown to be a key regulator of ribosome biogenesis. FMRP not only regulates rRNA volume (Seo et al. 2022) but as an RNA‐binding protein, FMRP interacts directly with snoRNA and regulates ribosomal RNA methylation which in turn controls rRNA folding and ribosome assembly. Moreover, a large number of FMRP‐associated proteins are involved in different stages of ribosome biogenesis (Richter and Zhao 2021). More recently, FMRP was also identified as a key translational regulator of ribosomal proteins (Seo et al. 2022). A direct link between reduced ribosome biogenesis and ageing has been described with small nucleolar size and reduced expression from the rDNA locus being a common feature across several distinct long‐lived models in different species (Tiku and Antebi 2018). We therefore propose that during normal ageing, DmGluRA activity limits the expression of FMRP relieving FMRP‐dependent inhibition of ribosomal biogenesis. Loss or inhibition of DmGluRA activity allows for increased FMRP expression which in turn inhibits ribosomal biogenesis leading to a longer lifespan. Thus, we have identified DmGluRA as a novel gerontological factor with life‐limiting activity in *Drosophila* and propose pharmacological inhibition of mGluR signalling as a novel geroprotection strategy.

## Author Contributions

C.G., A.O. and C.S. conceptualised the study. C.G., A.O. and C.S. designed the experiments. C.G., A.O., M.L. and C.S. conducted the experiments. C.G., A.O., I.P. and C.S. analysed the data. I.P. and C.G. analysed the RNA‐Seq data. C.G. and C.S. wrote the original manuscript. C.S. edited it.

## Conflicts of Interest

The authors declare no conflicts of interest.

## Supporting information


Appendix S1

## Data Availability

RNA‐seq data files are openly available in ArrayExpress at https://www.ebi.ac.uk/biostudies/arrayexpress, reference number E‐MTAB‐8307 (Slack 2023). All other data that support the findings of this study are available from the corresponding author upon reasonable request.
