# Supplementary material for: Genetic and Pharmacological Inhibition of Metabotropic Glutamate Receptor Signalling Extends Lifespan in Drosophila
Source: Aging Cell. 2025 Feb 12;24(5):e14500. doi: 10.1111/acel.14500 (PMC12073928; doi:10.1111/acel.14500)
Supplement: Supplementary file 1 — Appendix S1 [file ACEL-24-e14500-s001.pdf]

Table S1 - Increased FMRP expression upon loss of DmGluRA activity is required for lifespan extension, related to Figure 6

Genotype	Median Lifespan (days)	n Dead	n Censored	% Increase	p-value (log rank) vs control	Control
w^{Dah}	69	129	4			*
$DmGluRA^{[112b]}$	76	137	0	10	2.133E-30	
$dFmr1^{[392]}$	71	117	3	3	2.142E-10	
$dFmr1^{[392]}$;$DmGluRA^{[112b]}$	71	152	2	3	2.708E-08	

Cox Proportional Hazard (CPH) analysis

Details	Coefficient	Coefficient (estimate)	exp(coeff)	SE (coeff)	z	p
dead = 544	w^{Dah} vs $DmGluRA^{[112b]}$	-1.7472	0.1743	0.1408	-12.413	<2E-16
censored = 7	w^{Dah} vs $dFmr1^{[392]}$	-0.5648	0.5685	0.1296	-4.357	3.20E-05
Effect of $DmGluRA^{[112b]}$	w^{Dah} vs $dFmr1^{[392]}$: $DmGluRA^{[112b]}$	1.6802	5.3699	0.1855	9.057	<2E-16
mutation analysed using						
a proiri contrasts.						

A

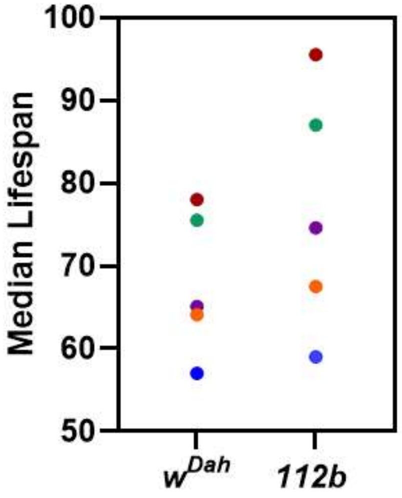

B

Experiment/Genotype	Median Lifespan (Days)	n Dead	n censored	% increase	p-value (log rank) vs control	Control
Experiment 1						
wDah	75.5	136	6	15	4.64727E-23	*
112b	87	117	19			
Experiment 2						
wDah	78	133	11	22	4.67583E-33	*
112b	95.5	151	7			
Experiment 3						
wDah	64.1	124	8	12	2.69596E-05	*
112b	71.6	110	12			
Experiment 4						
wDah	57	111	28	4	0.003455884	*
112b	59	117	27			
Experiment 5						
wDah	65.1	138	8	4	0.001247704	*
112b	67.5	146	6			

Supplementary Figure S1. Survival data for repeat independent experiments comparing *DmGluRA$^{[112b]}$* homozygous females to *w^{Dah}* controls.

(A) Median survival for *w^{Dah}* and *DmGluRA$^{[112b]}$* females across 5 independently replicated experiments. Colours indicate individual experiments. (B) Numbers of individuals (deaths and censors) and Log-rank *p*-values for the experimental data displayed in (A).

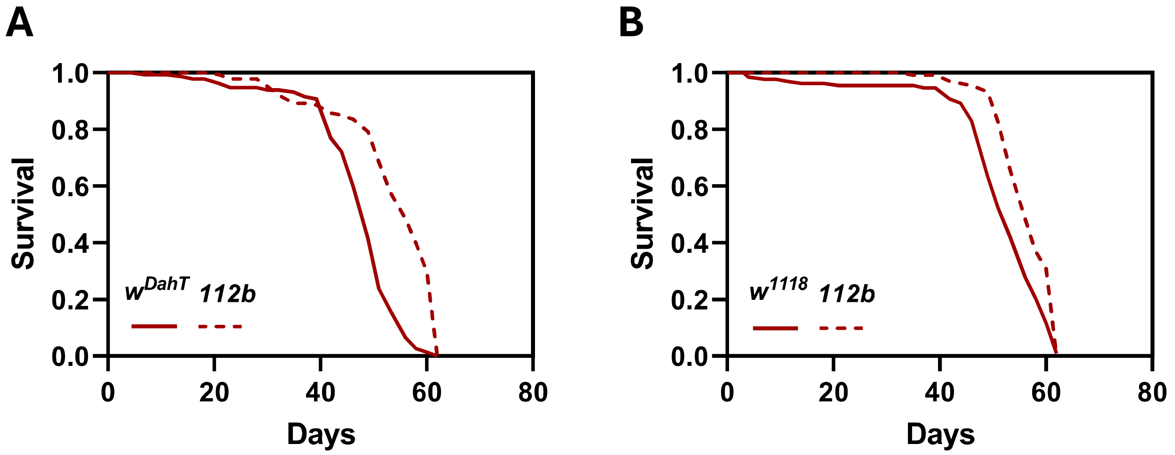

Supplementary Figure S2. Mutation of *DmGluRA* extends female lifespan irrespective of *Wolbachia* status or genetic background.

(A) Survival of female *DmGluRA*$^{[112b]}$ homozygous mutant flies after backcrossing into the *Wolbachia*-deficient w^{DahT} genetic background. (w^{DahT} controls: n = 116 dead/ 22 censored; *DmGluRA*$^{[112b]}$ homozygous mutants: n =127 dead/ 15 censored; p = 2.5x10^{-16}, Log-rank test). (B) Survival of female *DmGluRA*$^{[112b]}$ homozygous mutant flies after backcrossing into the w^{1118} genetic background. (w^{1118} controls: n = 123 dead/ 12 censored; *DmGluRA*$^{[112b]}$ homozygous mutants: n = 125 dead/ 15 censored; p = 1.6x10^{-6}, Log-rank test).

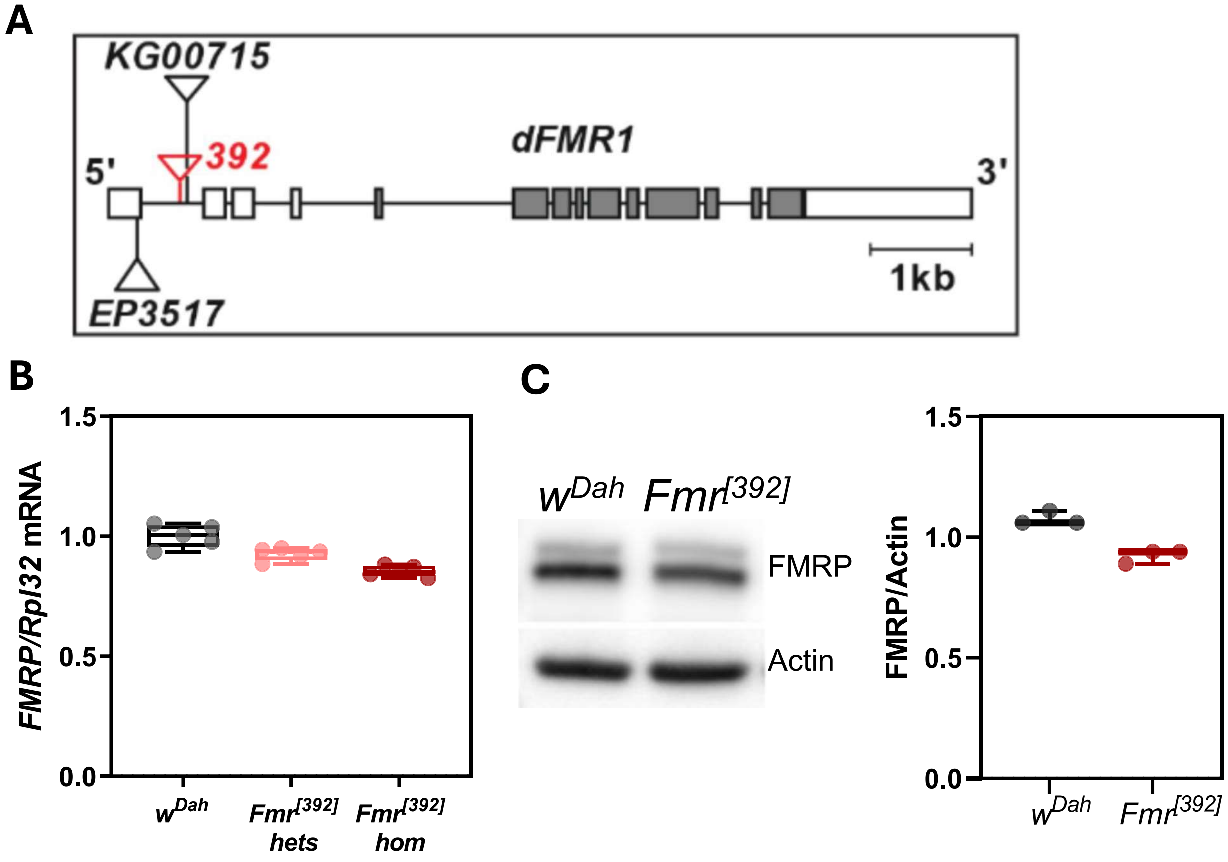

Supplementary Figure S3. Reduced *dFmr1* transcript and protein expression in *dFmr1*$^{[392]}$ mutants.

(A) Schematic of the *Drosophila dFmr1* locus showing the position of the *dFmr1*$^{[392]}$ p-element insertion. Black boxes represent exons and shaded boxes indicate the *dFmr1* coding sequence. (B) Quantitative RT-PCR analysis of *dFmr1* mRNA relative to *rpl32* in 10-day old female flies of the indicated genotypes (hets represent heterozygous and hom represents homozygous *dFmr1*$^{[392]}$ mutants). (n = 5 replicates of 15 female flies, $p<0.05$, ANOVA). (C) Representative Western blot showing FMRP protein expression in head extracts of 10-day old *dFmr1*$^{[392]}$ homozygote females compared to w^{Dah} controls. Box plots represent the relative expression of FMRP normalised to actin from three replicate Western blots (scaled to batch average). Data were analysed with a mixed effects linear model with sample batch as a random effect. The effect of genotype was significant (n=3, $p< 0.05$).
